# Induction of IL-25 secretion from tumour-associated fibroblasts suppresses mammary tumour metastasis

**DOI:** 10.1038/ncomms11311

**Published:** 2016-04-18

**Authors:** Shu-Yi Yin, Feng-Yin Jian, Yung-Hsiang Chen, Shih-Chang Chien, Mao-Chih Hsieh, Pei-Wen Hsiao, Wen-Hwa Lee, Yueh-Hsiung Kuo, Ning-Sun Yang

**Affiliations:** 1 Agricultural Biotechnology Research Center, Academia Sinica, Taipei 115, Taiwan; 2 The Experimental Forest Management Office, National Chung Hsing University, Taichung 402, Taiwan; 3 Department of Surgery, Wan-Fang Hospital, Taipei 116, Taiwan; 4 China Medical University, Taichung 404, Taiwan; 5 Department of Chinese Pharmaceutical Sciences and Chinese Medicine Resources, China Medical University, Taichung 404, Taiwan; 6 Department of Biotechnology, Asia University, Taichung 413, Taiwan

## Abstract

Tumour-associated fibroblasts (TAFs), as a functionally supportive microenvironment, play an essential role in tumour progression. Here we investigate the role of IL-25, an endogenous anticancer factor secreted from TAFs, in suppression of mouse 4T1 mammary tumour metastasis. We show that a synthetic dihydrobenzofuran lignan (Q2-3), the dimerization product of plant caffeic acid methyl ester, suppresses 4T1 metastasis by increasing fibroblastic IL-25 activity. The secretion of IL-25 from treated human or mouse fibroblasts is enhanced *in vitro*, and this activity confers a strong suppressive effect on growth activity of test carcinoma cells. Subsequent *in vivo* experiments showed that the anti-metastatic effects of Q2-3 on 4T1 and human MDA-MD-231 tumour cells are additive when employed in combination with the clinically used drug, docetaxel. Altogether, our findings reveal that the release of IL-25 from TAFs may serve as a check point for control of mammary tumour metastasis and that phytochemical Q2-3 can efficiently promote such anticancer activities.

## Abstract (web summary)

Interleukin-25 has been reported to have anticancer activity with very little effect on non-malignant cells. Here, the authors show that a synthetic phytochemical can be used to induce the secretion of Interleukin-25 from tumour associated fibroblasts resulting in impaired tumour metastasis.

## Graphical abstract

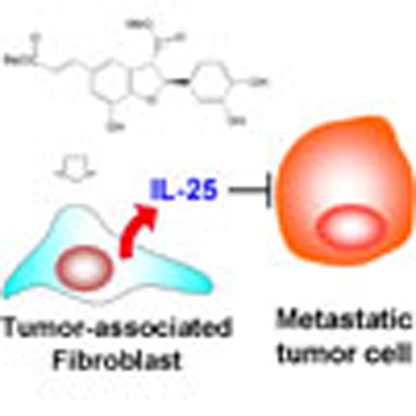

Breast cancer is the most common malignancy in women worldwide and the second leading cause of cancer mortality^1^. In these patients, it is not the primary tumour, but its metastases to distant sites that are the main cause of death. Clinical surgery via resection of the malignant primary tumour is still the routine primary treatment for breast cancer patients^2^. Increasing evidence suggests that tumour cells are conditioned by their tissue-microenvironments at primary and secondary sites for growth and metastasis. The challenge now is therefore to prevent or suppress metastasis of cancer cells from the tumour-associated microenvironment into target tissues^3^. The tumour microenvironment has been described as a tumour stroma or premetastatic/metastatic niche that can promote metastasis and therapy resistance^3^,^4^,^5^. On the other hand, tumour-associated stromal cells can also produce tumour suppressor factors, such as nucleoside diphosphate kinase A (NME1)^6^, Kangai 1 (KAI1/CD82)^7^,^8^ and IL-25 (ref. 9), in the tumour microenvironment, and this can restrict the development or metastasis of breast cancers. Research into molecular agents that can confer a strong stimulatory effect on the expression of potent metastasis suppressor molecules is one direction that may lead to new cancer therapies^10^,^11^,^12^.

Lignans, as widespread plant natural products, have a broad variety of chemical structures and exhibit a large range of biological activities^13^. A series of synthetic dihydrobenzofuran lignans, obtained by biomimetic oxidative dimerization of caffeic and/or ferulic acid methyl ester followed by derivatization reactions have been shown to exhibit potent antiangiogenic activity^14^. Among these synthetic compounds, methyl(*E*)-3-[2-(3,4-dihydroxyphenyl) -7-hydroxy-3-methoxycarbonyl-2,3-dihydro-1-benzofuran-5yl]-prop-2-enoate (Q2-3) has been shown to exhibit a significant anti-proliferation effect on various human cancer cell lines, including Jurkat, K562 and MCF-7 cells^15^. Although *in vitro* study has indicated a specific effect of Q2-3 on cytotoxicity or G2/M cell cycle arrest in Jurkat cells^15^, the anti-metastatic effect of this synthetic compound *in vivo* has not been addressed in previous study. In this study, we first investigated whether Q2-3 and some other selected lignans could interfere with mammary tumour metastasis in a tumour resection mouse model. As compared with other tested lignans, Q2-3 conferred a significant anti-metastatic effect on test mammary tumours. In particular, we investigated whether specific cellular mechanisms of Q2-3 action, including tumour-associated fibroblast (TAF) activities in the tumour microenvironment, are associated with such bioactivity. We thus mimicked an *in vitro* mammary tumour microenvironment by using a three-dimensional (3D) cell co-culture system to assess the regulatory effect of Q2-3 on the expression of specific cytokines and innate immune cell activities in both human and mouse TAFs.

Interleukin-25 (IL-25/IL-17E) was recently reported to confer high anticancer activity, with little or no effect on non-malignant cells^9^. The apoptotic activity of IL-25 was shown to be mediated by differential expression of its receptor, IL-25R, which was found to be expressed at high levels in tumours from patients with poor prognoses, but at low levels in non-malignant breast tissues^9^. This finding suggests that targeting the IL-25 signalling pathway may offer a novel therapeutic approach for advanced breast cancers. In this study, our findings also indicate that the stromal fibroblasts in the mammary tumour microenvironment can express IL-25 which can in turn mediate an anti-metastatic effect on the companion tumour cells. In addition, Q2-3 can greatly enhance such endogenous activity of TAFs and result in a potent anti-metastatic effect against the surrounding mammary carcinoma cells. The possible implications and application of our findings, in terms of the mechanistic regulation of tumour microenvironments and potential clinical inference with tumour metastasis using specific phytochemicals as IL-25 agonist, are discussed.

## Results

### Q2-3 confers a specific toxicity on mammary carcinoma cells

**Figure 1:**
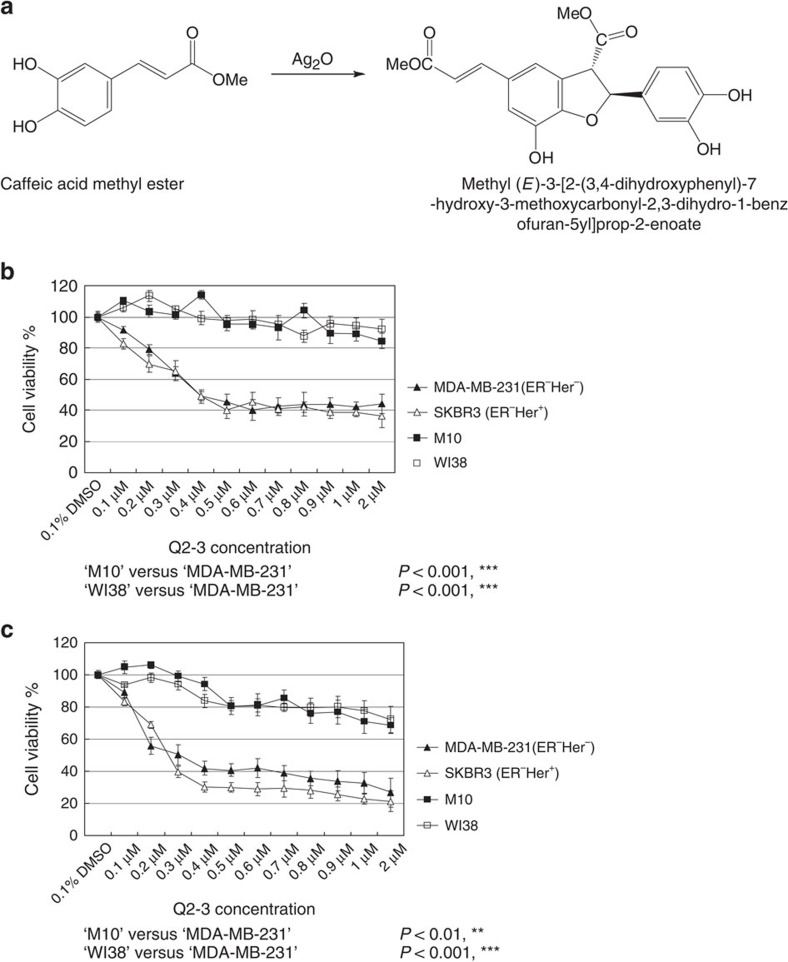
Dihydrobenzofuran lignan (Q2-3) exhibits a specific cytotoxic effect on breast tumour cells. (**a**) Chemical reaction formula for synthesis of Q2-3 compound (methyl (*E*)-3-[2-(3,4-dihydroxyphenyl)-7-hydroxy-3-methoxycarbonyl-2,3-dihydro-1-benzofuran-5yl]prop-2-enoate) from two caffeic acid methyl ester molecules. (**b**,**c**) MTT assays for cytotoxic effect of Q2-3 on SKBR3 (ER^−^Her^+^ human breast cancer cell line), MDA-MB-231 (ER^−^Her^−^ human breast cancer cell line), M10 (human normal epithelial cell line) and WI38 (human lung fibroblast cell line). 2 × 10^4^ cells were seeded and compared for their growth activity after treatment with Q2-3 for 24 or 72 h. The beginning cytotoxic dosage of Q2-3 was detected at 0.3 μM, with >50% kill rate on SKBR3 and MDA-MB-231 cells. Data are reported as mean±s.d. (*n*=4). **P<0.01, ***P<0.001 (Two-tailed *t*-test). Similar results were obtained from three independent experiments.

To evaluate the anticancer effect of methyl (*E*)-3- [2-(3,4-dihydroxyphenyl)-7-hydroxy-3-methoxycarbonyl-2,3-dihydro-1-benzofuran-5yl]prop-2-enoate, denoted as Q2-3, an organic synthesis reaction was needed^13^,^14^. This was achieved by the dimerization of methyl caffeic acid as catalysed in the presence of silver oxide (Fig. 1a). The yield of Q2-3 after the indicated reaction was ∼32%. We compared the cytotoxic activities of this synthetic compound on various mammary epithelial cells and fibroblasts. The compound was ∼96% pure as a single Q2-3 chemical, as determined by elemental composition analysis and HPLC (see Methods). The effects of Q2-3 on cell viability were tested on four different cell lines, M10, WI38, SKBR3 and MDA-MB-231, by treating cells with varying Q2-3 concentrations for 24 h (Fig. 1b) and 72 h (Fig. 1c). As compared with the control (0.1% dimethyl sulphoxide (DMSO)) group cells, Q2-3 effectively suppressed the growth (≥60% inhibition) of human mammary tumour cells (SKBR3 and MDA-MB-231 cells) at a relatively low concentration (≥0.5 μM). In contrast, normal human mammary epithelial (M10) and fibroblast (WI38) cell lines showed a much higher resistance to treatment with Q2-3, exhibiting≥80% viability at 2 μM (Fig. 1b,c). This result is strongly supported by data collected from more than 10 serial concentrations tested at two different time points (24 and 72 h). This result indicates a high cytotoxicity of Q2-3 on mammary tumour cells. In contrast, normal human mammary epithelial cells and fibroblasts exhibited a much higher level of tolerance for Q2-3 treatment.

### Q2-3 can suppress metastasis of mammary tumour cells

**Figure 2:**
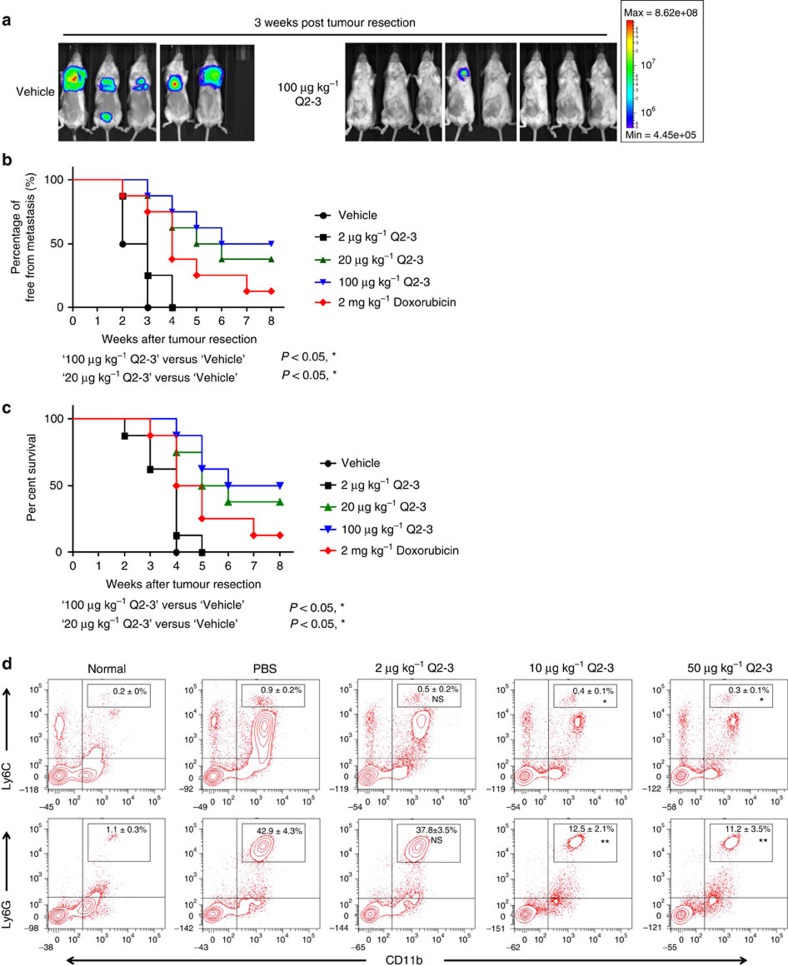
*In vivo* treatment of Q2-3 significantly suppresses the metastasis of mouse (4T1) mammary tumour cells after a tumour resection process. (**a**) Representative bioluminescent images of tumour-bearing mice (*n*=8 per group) after *in vivo* treatment with PBS (0.1% DMSO in saline), doxorubicin (2 mg kg^−1^) or different dosages of Q2-3, after resection of the orthotopic primary tumours. In the PBS-treated group (vehicle), three mice died before 3 weeks post tumour resection. The red signal represents the highest level on the colorimetric scale. (**b**) Quantification of tumour metastasis by measuring luciferase activity in photons s^−1^ cm^−2^ sr^−1^ in mice revealed along the indicated time course (*n*=8 per group). (**c**) Survival of test mice after different treatments. *P*<0.05, were obtained between the control (DMSO) and Q2-3-treated (20 or 100 μg kg^−1^) groups (Kaplan–Meier results were analysed by log-rank test). (**d**) Effect of Q2-3 (2, 20 or 50 μg kg^−1^) on the population change of monocytic (CD11b^+^Ly6C^+^) and granulocytic (CD11b^+^Ly6G^+^) MDSCs in lung tissues of test mice were quantified by flow cytometry analysis, and were compared with MDSC populations in PBS group. Data are reported as mean±s.d. (*n*=3). *P<0.05; **P<0.01; NS, not significant (two-tailed *t*-test). Similar results were obtained from three independent experiments.

To investigate the anti-metastatic effects of Q2-3, transgenic luciferase-expressing mouse 4T1-Luc2 cells were injected into the mammary fat pad of test mice. At 15 days post tumour cell implantation, *in situ* 4T1 tumours were carefully removed by a surgical resection process. Tumour metastatic activity and survival time of control and Q2-3-treated mice were examined and compared over the following 8 weeks (Fig. 2a–c). By detecting the luminescent activity of 4T1-Luc2 cells as an indication of tumour metastasis, Q2-3 treatment (≥20 μg kg^−1^) was found to significantly suppress the metastasis of 4T1 cells to the lung (Fig. 2a). In addition, treatment with Q2-3 at a relatively low dosage (>20 μg kg^−1^) had much higher anti-metastatic activity than treatment with doxorubicin (2 mg kg^−1^) (Fig. 2b), a drug which is used clinically for the treatment of human breast cancers^16^,^17^. Q2-3 treatment also consistently, significantly increased the survival rate of tumour-resected mice (Fig. 2c). These results show that *in vivo* administration of Q2-3 can efficiently prevent mammary tumour metastasis after a tumour resection process.

The importance of myeloid derived suppressor cells (MDSCs) in regulation of tumour growth has been well documented^18^,^19^. A drastic accumulation and activation of MDSCs is also recognized as an important pathologic feature of tumour progression and this can be readily observed in the 4T1 tumour resection model, with which the populations of both monocytic and granulocytic MDSCs in metastatic tissues could be detected at high abundance at 3-4 weeks post tumour resection. Therefore, we also evaluated the suppressive effect of Q2-3 on the *in vivo* expansion activity of MDSC populations, that is, for both of the monocytic (CD11b^+^Ly6C^+^) and granulocytic (CD11b^+^Ly6G^+^) subsets of MDSCs. The populations of these two MDSC cell types in lung tissues of test mice were analysed and compared at 3 weeks post tumour resection. Compared with the PBS (vehicle control)-treated mice, both CD11b^+^Ly6C^+^ and CD11b^+^Ly6G^+^ MDSC populations in Q2-3-treated (10 μg kg^−1^) mice were significantly decreased from 0.9% to 0.4% and from 42.9% to 12.5%, respectively (Fig. 2d). This result is consistent with the data shown in Fig. 2a–c, and together supports the observed anti-metastatic effect of Q2-3 on mammary tumour cells.

### Q2-3 treatment induces IL-25 expression in lung fibroblasts

**Figure 3:**
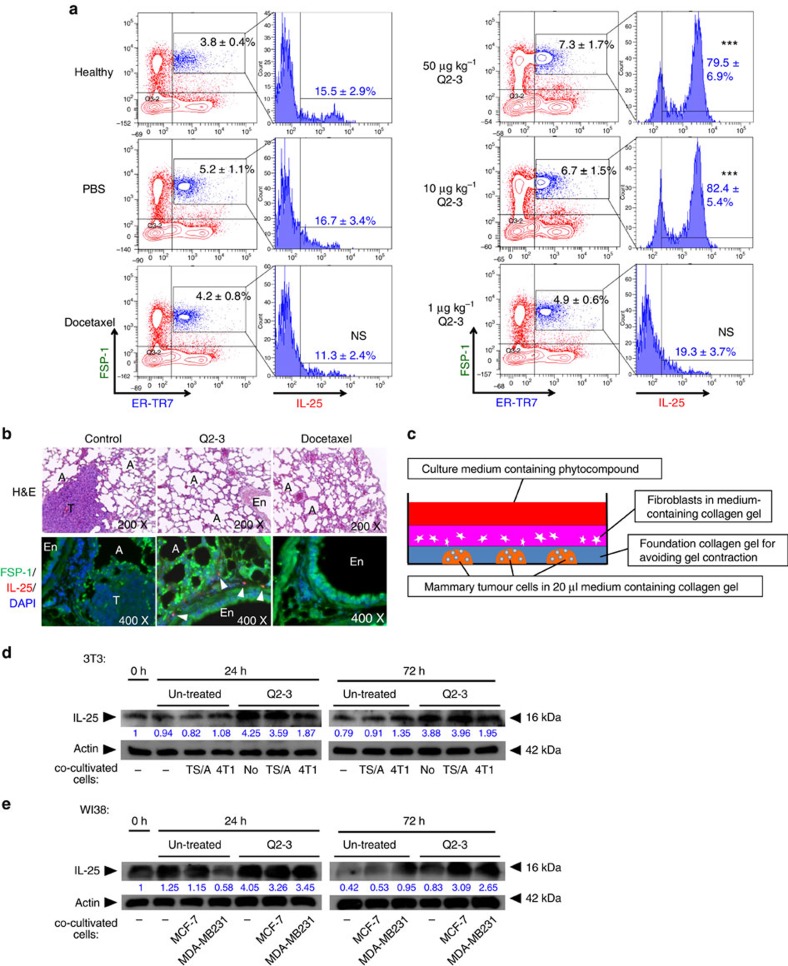
Q2-3 treatment significantly upregulates IL-25 expression in tumour-associated fibroblasts, as evaluated by 3D cell co-culture system. (**a**) Effect of Q2-3 on population change of FSP-1^+^ER-TR7^+^ cells and their IL-25 expression level in lungs of test mice, quantified using flow cytometry. The percentage of IL-25^+^ fibroblasts in gated FSP-1^+^ER-TR7^+^ cells were compared with corresponding IL-25^+^ fibroblast population in PBS group. Data are reported as mean±s.d. (*n*=3). *P<0.05; ***P<0.001; NS, not significant (two-tailed *t*-test). FSP-1, fibroblast specific protein-1; ER-TR7, Erasmus University Rotterdam-thymic reticulum-7.IF staining; lung tissues of control (0.1% DMSO in PBS), Q2-3-treated and docetaxel-treated mice were collected at 21 days post tumour resection and stained for the presence of IL-25-expressing cells (red and indicated with arrowheads), FSP-1 (green) and DAPI (blue). T, tumour; A, alveoli. (**b**) IF staining, lung tissues of control (0.1% DMSO in PBS), Q2-3-treated and docetaxel-treated mice were collected at 21 days post tumour resection and stained for the presence of IL-25-expressing cells (red and indicated with arrowheads), FSP-1 (green) and DAPI (blue). T, tumour; A, alveoli. (**c**) Construction of 3D cell co-culture system for mammary tumour cells and tumour-associated fibroblasts. Western blot analyses of the expression of (**d**) mouse IL-25 in 3T3 fibroblasts and (**e**) human IL-25 in WI38 fibroblasts, in response to Q2-3 treatments in 3D culture. Some fibroblasts in the upper layer were co-cultured with mammary tumour cells as indicated. The expression level of IL-25 in 3T3 and WI38 fibroblasts were quantified and normalized using ImageJ software. Fold changes of IL-25 expression level in test samples were normalized with the value in fibroblasts of the 0 h group and were indicated by the number labelled in blue. β-Actin served as an internal control. Similar results were obtained from three independent experiments.

In previous *in vitro* studies, Q2-3 was shown to confer high toxicity to various types of mammary tumour cells^14^,^15^. However, in our current *in vivo* experiments, Q2-3 administration only slightly suppressed the growth of *in situ* caricinoma (Supplementary Fig. 1). By taking these results together with the potent anti-metastatic effect of Q2-3 (Fig. 2), we hypothesize that *in vivo* administration of Q2-3 may also confer a regulatory effect on the target metastatic tissues. To characterize the physiological significance of the modulatory activity of Q2-3, we analysed the expression of several secreted cytokines *in vivo* in lung tissue of test mice. The change in the expression level of IL-25 upon Q2-3 treatment was particularly striking. By comparison, we found that Q2-3 administration (100 μg kg^−1^) conferred a readily detectable stimulatory effect on IL-25 activity in lung fibroblasts, which were found to mainly surround the main pulmonary artery and vein tissue (Fig. 3b). In lung tissues of control and docetaxel-treated mice, little or no IL-25 expression was detected in lung fibroblasts. This result suggests that Q2-3-induced IL-25 expression is specifically expressed in the fibroblasts of the lung tissue microenvironment, which is not a pharmacological target of conventionally used anticancer drugs. To quantify the change in cell population of IL-25-expressing lung fibroblasts in response to Q2-3 treatment, populations of FSP-1^+^ER-TR7^+^ cells in test mouse lung tissues were quantified and compared for their IL-25 expression level at 3 weeks post tumour resection. Compared with the PBS (vehicle control)-treated mice, the cell population of IL-25^+^ fibroblasts (FSP-1^+^ER-TR7^+^IL-25^+^ cells) in Q2-3-treated mice was found to be drastically increased from 16.7 to 79.5% (Fig. 3a). In addition, the total FSP-1^+^ER-TR7^+^ fibroblast population in Q2-3-treated mice was also detected to be increased from 5.2 to 7.3%, in a dose-dependent manner (Fig. 3a). In contrast, treatment with docetaxel, a drug tested in parallel as a reference, did not increase the quantity and the IL-25 expression level of FSP-1^+^ER-TR7^+^ cells in lungs of test mice. This result is consistent with the data shown in Fig. 3a, and further quantifies and supports the finding that Q2-3 stimulates pulmonary fibroblasts *in vivo*.

To further study the specific effect of Q2-3 on fibroblasts in the tumour microenvironment, a three-dimensional (3D) cell co-culture system was employed to mimic the *in vivo* mammary tumour microenvironment (Fig. 3c). In this system, high density of mouse or human mammary carcinoma cells were mixed in medium-containing collagen (1 × 10^5^ cells per 20 μl medium-containing collagen solution) and dropped onto the culture substratum (4 colonies per well). As described in Methods, 1 × 10^6^ mouse (3T3 cells) or human (NHDF and WI38 cells) fibroblasts were grown in the upper layer of 3D collagen gel and co-cultured with or without test mammary tumour cells in the lower layer. With this artificial reconstruction approach, we compared the expression level of IL-25 in mouse and human fibroblasts, alone or in co-cultivation with test mammary tumour cells. In addition, this 3D setup also allowed us to study the effect of Q2-3, which was supplemented into the test culture media. In comparison, expression of IL-25 in both mouse (3T3) and human (WI38) fibroblasts were increased after Q2-3 treatment for 24 or 72 h (Fig. 3d,e). This result suggests that Q2-3 can significantly upregulate IL-25 expression in both mouse and human fibroblasts.

### Fibroblasts-secreted IL-25 suppresses growth of tumour cells

**Figure 4:**
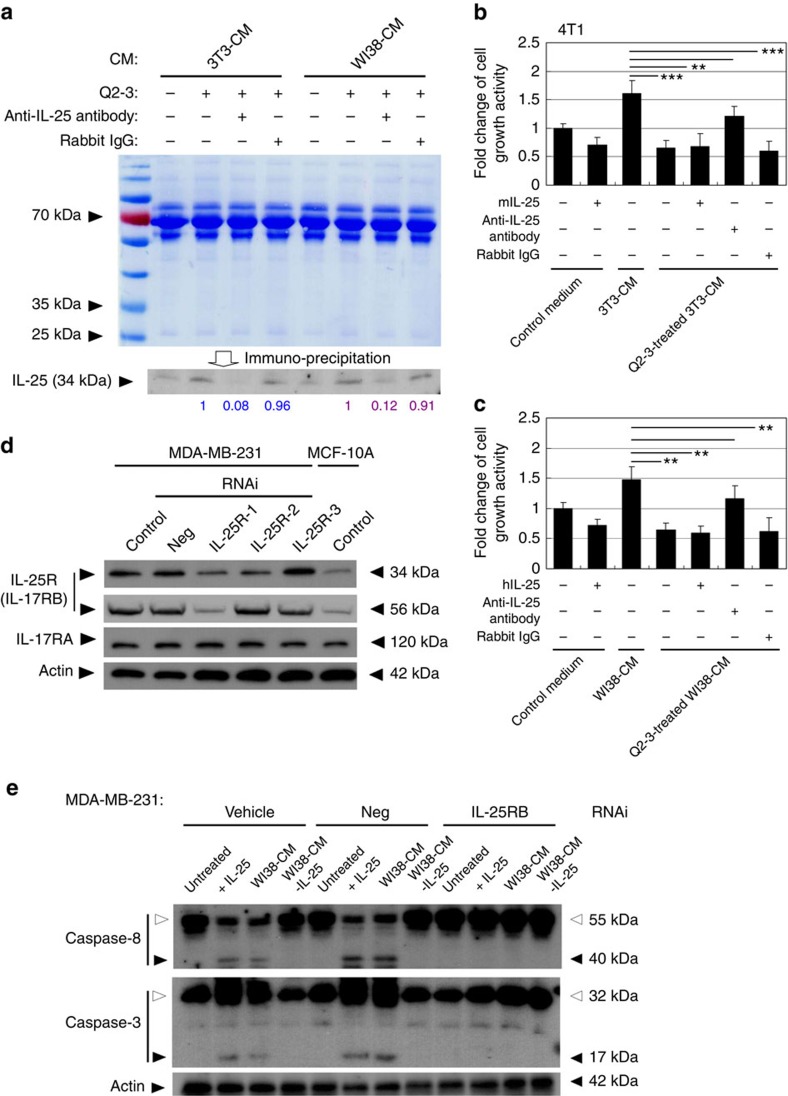
IL-25 secreted by Q2-3-treated fibroblasts suppresses the growth of mammary tumour cells. (**a**) Western blot analysis of the IL-25 secretion activity of mouse (3T3) and human (WI38) fibroblasts in response to Q2-3 treatment. Different fibroblast-conditioned media (CM), including 3T3-CM and WI38-CM, were collected from the 3D cultures and were stained with coomassie blue, revealing that the total protein level in tested CM was normalized. Aliquots of 3T3-CM and WI38-CM were immunodepleted for IL-25. Rabbit IgG (isotype control) was used as a negative control. Amounts of IL-25 (relative staining intensity) were further normalized with the value detected for Q2-3-treated 3T3-CM (blue) or Q2-3-treated WI38-CM samples (purple). (**b**) Reduction in cytotoxicity of 3T3-CM on 4T1 cells after immunodepletion of IL-25. The control (fresh) medium, 3T3-CM, Q2-3-treated 3T3-CM, Q2-3-treated 3T3-CM with added IL-25 protein, Q2-3-treated 3T3-CM with the immunodepletion of IL-25 and Q2-3-treated 3T3-CM with control IgG-mediated immunodepletion, were compared for their suppressive effect on growth of 4T1 cells. (**c**) Reduction in cytotoxicity of WI38-CM on MDA-MB-231 after immunodepletion of IL-25. Similarly, WI38-CM with added IL-25 protein, WI38-CM with the immunodepletion of IL-25, or Q2-3-treated WI38-CM with control IgG-mediated immunodepletion, were compared for their effect on growth of MDA-MB-231 cells. The growth activity of 4T1 cells or MDA-MB-231 cells was determined using MTT assay at 5 days post cultivation, and was normalized to the control group (with fresh medium only). Data are reported as mean±s.d. (*n*=3). *P<0.05; **P<0.01; ***P<0.001 (two-tailed Student's *t*-test). (**d**) Western blot analysis of IL-17RA and IL-17RB (IL-25R) expression in MDA-MB-231 and MCF-10A cells. Knock down of human IL-25R (both 56 and 33 kDa molecules) expression in MDA-MB-231 cells was performed with three designed siRNAi treatments. Neg, negative control RNAi. (**e**) Western blot analysis of the cleavage of caspases 8 and 3 in MDA-MB-231 cells. MDA-MB-231 cells were treated by control-, Neg RNAi- or IL-25R RNAi, for 48 h. Some cells in each test group were cultivated with recombinant human IL-25 (200 ng ml^−1^), WI38-CM, or WI38-CM with the immunodepletion of IL-25, for another 24 h. White arrowheads, cleaved protein. Black arrowheads, uncleaved protein. β-Actin served as an internal control. Similar results were obtained from three independent experiments.

To characterize and analyse the possible suppressive effect of fibroblast-secreted IL-25 on growth activity of mammary tumour cells, the levels of secreted IL-25 protein in conditioned media of test mouse and human fibroblasts were collected and compared by using an anti-IL-25 antibody-mediated immunoprecipitation approach. Before immunoprecipitation, aliquot samples of conditioned medium from Q2-3-treated fibroblasts, including 3T3 fibroblast-conditioned media (3T3-CM) and WI38 fibroblast-conditioned media (WI38-CM), were immunodepleted for IL-25. In this test, anti-rabbit IgG antibody (isotype control) was used as a negative control for immunodepletion. The quantity of IL-25 in each conditioned medium and the efficiency of immunodepletion for IL-25 were then assessed by immunobloting analysis (Fig. 4a). In comparison, the levels of secreted IL-25 in the Q2-3-treated fibroblast-conditioned media were found to be significantly higher than that in the untreated fibroblast-conditioned media (Fig. 4a). Importantly, this result also demonstrates that most secreted IL-25 (∼90%) in both human and mouse Q2-3-treated fibroblast-conditioned media could be immunodepleted by using anti-IL-25 antibody (Fig. 4a). Only a very small fraction (4 to 9% decrease) of nonspecific protein binding was detected for the isotype control antibody, showing high specificity and efficiency of the used anti-IL-25 antibody. The conditioned media immunodepleted for IL-25 from cultivation of mouse and human fibroblasts were also employed to culture 4T1 and MDA-MB-231 tumour cells, respectively. In this test, fresh conditioned media were applied every 24 h for 5 days. 4T1 cells cultured with 3T3-CM showed much higher growth activity than cells cultured with fresh medium only (Fig. 4b). This result is consistent with that obtained from MDA-MB-231 cells (Fig. 4c) and taken together these data suggest that fibroblasts can release important cellular and molecular factors for tumour cell expansion. In addition, exogenously added IL-25 protein (100 ng ml^−1^ for human and mouse IL-25 recombinant protein) decreased the growth activity of test 4T1 and MDA-MB-231 cells. In agreement, 4T1 cells cultured with Q2-3-treated 3T3-CM (Fig. 4b) or MDA-MB-231 cells cultured with Q2-3-treated WI38-CM (Fig. 4c) also showed relatively decreased cell growth activity, as compared with IL-25 protein-treated tumour cells. In contrast, depletion of IL-25 attenuated the growth-suppressive effect of Q2-3-treated fibroblasts CM on mouse (Fig. 4b) and human (Fig. 4c) mammary tumour cells. This attenuation was not observed by the use of isotype IgG antibody. Taken together, our results suggest that the Q2-3-induced IL-25 secretion from fibroblasts plays a critical role in suppressing the growth activity of metastatic mammary tumour cells.

To examine whether the growth-suppression activity of fibroblast-secreted IL-25 protein was mediated by IL-25R (IL17RB) signalling in mammary tumour cells, we knocked down the expression of IL-25R in metastatic mammary carcinoma cells (MDA-MB-231 cells) using IL-25R-specific small interfering RNA (siRNA). To test the knockdown efficiency of three designed IL-25R siRNAs, we screened for IL-25R expression in MDA-MB-231 cells (Fig. 4d), and compared it with that in MCF-10A cells, a non-malignant and oestrogen receptor (ER)-negative breast cancer cell line with low levels of expression of IL-25R (Fig. 4d). The negative control siRNA (non-targeting siRNA) treatment did not have a significant effect on expression of IL-25R. Among the three tested siRNAs, treatment with IL-25R-1 siRNA exhibited the highest knockdown efficiency for the expression of both IL17RB isoforms (33 and 56 kDa) in treated MDA-MB-231 cells (Fig. 4d). In addition, none of the IL-25RB siRNA treatments resulted in a detectable change in the expression level of IL-17RA (Fig. 4d), another component of the IL-25R heterodimer. Altogether, the IL-25R-1 siRNA preparation was therefore chosen for subsequent experiments. Treatment with IL-25 (100 ng ml^−1^) or Q2-3-treated WI38 conditioned medium (Q2-3-WI38-CM) resulted in the cleavage of caspases 8 and 3 in MDA-MB-231 cells (Fig. 4e), indicating the activation of apoptosis. Consistent with the result in Fig. 4c, depletion of IL-25 from Q2-3-WI38-CM also rescued test tumour cells from caspase cleavage (Fig. 4e). In contrast, in MDA-MB-231 cells treated with IL-25R siRNA, treatment with IL-25 or Q2-3-WI38-CM was not able to induce the cleavage activity of caspases 8 and 3 (Fig. 4e). These results suggest that the IL-25 secreted by Q2-3-treated WI38 fibroblasts can effectively induce IL25R-mediated cell apoptosis, which has been indicated to be a strong death signal that is specific to breast cancer cells^9^.

### Q2-3 induces anti-metastatic effect through IL-25 activities

**Figure 5:**
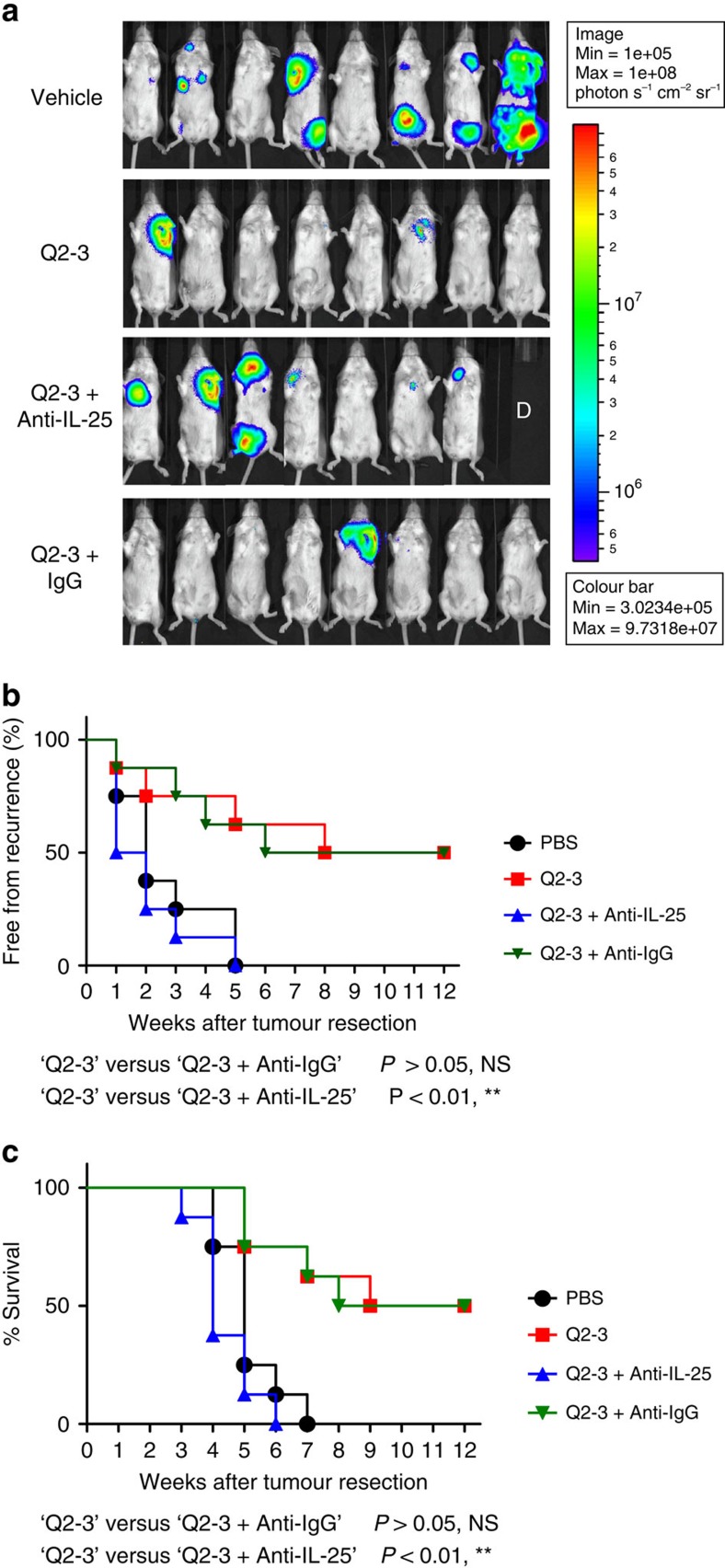
*In vivo* neutralization of IL-25 activity can effectively invalidate the anti-metastatic activity of Q2-3. (**a**) Representative bioluminescent images of tumour-resected mice (*n*=8 per group) after *in vivo* treatment with PBS (0.1% DMSO in saline), Q2-3 (100 μg kg^−1^; 3 injections per week), anti-mouse IL-25 Ab (100 μg per mice; 2 injections per week), Q2-3+IL-25 Ab, or Q2-3+isotype IgG, at 3 weeks post tumour resection. The label ‘D' in the photograph denotes the mice died before 3 weeks post tumour resection. (**b**) Quantification of tumour metastasis by measuring luciferase activity photons s^−1^ cm^−2^ sr^−1^ in mice, as revealed along the indicated time course (12 weeks). (**c**) Survival of test mice after different treatments. NS, no significant difference between the ‘Q2-3' and ‘Q2-3+Anti-IgG' groups. **P<0.01, was obtained between the ‘Q2-3' and ‘Q2-3+Anti-IL-25' groups (Kaplan–Meier results were analysed by log-rank test). Similar results were obtained from two independent experiments.

To address whether IL-25 expression plays a key role in the anti-metastatic activity of Q2-3 on mammary tumour cells, we further employed an antibody-neutralization approach to deplete the *in vivo* IL-25 activity in the same 4T1 tumour resection model. Again by detecting the luminescent activity derived from transgenic 4T1-Luc2 cells in test mice, co-treatment of mice with Q2-3 (100 μg kg^−1^) and anti-mouse IL-25 antibody (100 μg per injection per mice), unlike the anti-metastatic effect detected for the ‘Q2-3 treatment only' group, resulted in full metastatic activity as observed for the control (PBS) group. In contrast, by using an irrelevant anti-IgG antibody preparation for this antibody-depletion test, the Q2-3 effect on anti-metastasis was sustained virtually in total (Fig. 5a,b). These results show that IL-25 obviously plays a central role in anti-metastatic activity of Q2-3. Consistently, the mice in the co-treatment group (Q2-3+Anti-IL-25) also exhibited a survival rate that was decreased to a similar level to that of the control (PBS) group, as compared with the mice of the Q2-3-treated group (Fig. 5c). Interestingly, even *in vivo* administration of anti-mouse IL-25 antibody was repeatedly found to detectably promote 4T1 metastasis, as compared with that of the control tumour-resected mice (Fig. 5a,b). This finding suggests that the presence or expression of endogenous IL-25 caused weak suppression of tumour metastasis in the untreated, tumour-resected mice. Altogether, these results suggest that the *in vivo* anti-metastatic effect of Q2-3 is mediated by endogenous IL-25 activity.

**Figure 6:**
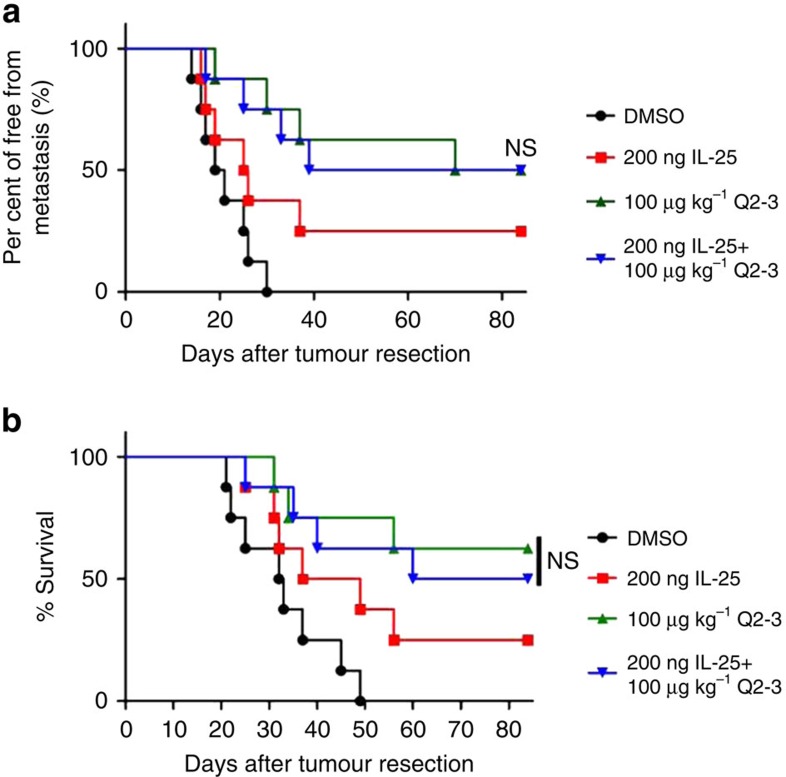
*In vivo* treatment of Q2-3 confers comparable anti-metastatic activity with IL-25 administration. (**a**) Tumour-resected mice (*n*=8 per group) were treated with PBS (0.1% DMSO in saline), IL-25 (200 ng per mice), Q2-3 (100 μg kg^−1^) or co-treated with IL-25 and Q2-3 for 3 weeks. Quantification of tumour metastasis by measuring luciferase activity in photons s^−1^ cm^−2^ sr^−1^ in mice revealed along the indicated time course. (**b**) Survival of test mice after different treatments. NS, no significant difference between the Q2-3 and co-treatment groups (Kaplan–Meier results were analysed by log-rank test). Similar results were obtained from three independent experiments.

In this study, the effect of IL-25 treatment *in vivo* was also compared for its additive versus overlapping effect. The co-treatment of mice with Q2-3 (100 μg kg^−1^) and IL-25 (10 μg kg^−1^) was found to confer a similar, rather than additive effect on anti-metastatic activity, as detected in a Q2-3 treatment only mouse group (Fig. 6a). Consistently, the mice in the co-treatment group also showed a survival rate increased by a similar level to the Q2-3-treated group, as compared with the mice in the untreated group (Fig. 6b). In other words, the anti-metastatic effect of Q2-3 *in vivo* can be effectively substituted by the administration of exogenous IL-25. These results also support the critical role of IL-25 in the anti-metastatic effect of Q2-3.

### Anti-metastatic effect of Q2-3 is synergistic to docetaxel

**Figure 7:**
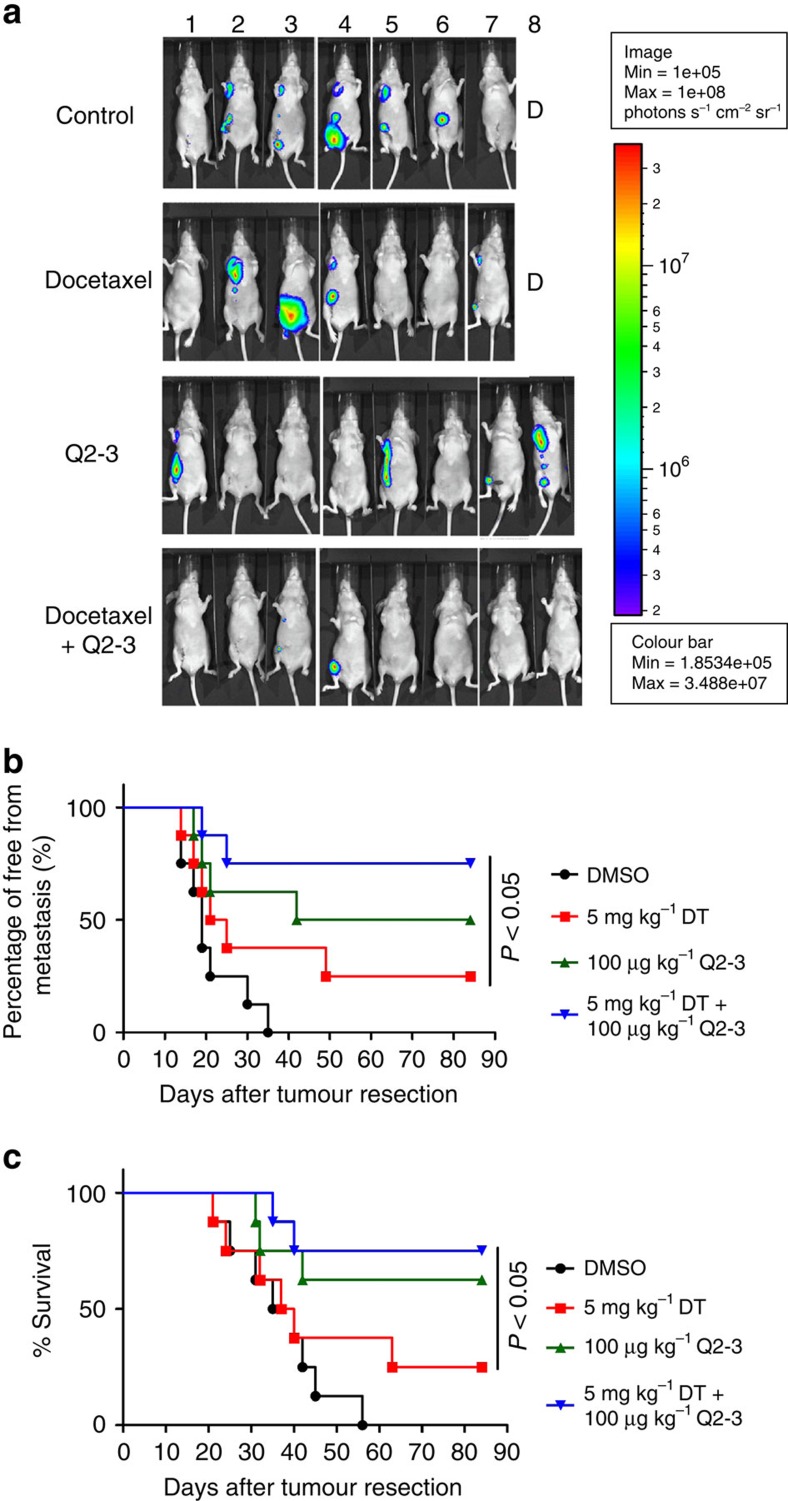
Q2-3 administration can confer a complementary anti-metastatic effect on human cancer cells (MDA-MB-231), when used in combination with docetaxel. (**a**) Representative bioluminescent images of MDA-MB-231 tumour-bearing nude mice (*n*=8 per group) after *in vivo* treatment with PBS (0.1% DMSO in saline), Q2-3 (100 μg kg^−1^), docetaxel (5 mg kg^−1^) or co-treatment with docetaxel and Q2-3 for 3 weeks, after resection of the orthotopic primary tumours. In PBS-treated (control) and docetaxel-treated groups, one mice was died before 3 weeks post tumour resection. (**b**) Quantification of tumour metastasis by measuring luciferase activity in photons s^−1^ cm^−2^ sr^−1^ in mice revealed along the indicated time course. (**c**) Survival of test mice after different treatments. *P*<0.05, were obtained between the docetaxel- and co-treated mice (Kaplan–Meier results were analysed by log-rank test). Similar results were obtained from two or three independent experiments.

To further evaluate whether Q2-3 can confer a complementary or additive therapeutic effect on the suppression of tumour metastasis when used in combination with other clinically used anticancer drugs, we assessed effect of Q2-3 plus docetaxel, a drug commonly used for the treatment of human breast cancer, in suppressing the metastatic activities of human MDA-MB-231-Luc2 cells in nude mice (Fig. 7). By detecting the luminescent activity of MDA-MB-231-Luc2 cells in test mice after resection of primary mammary tumour tissues *in situ* (Fig. 7a), we showed that combination treatment with Q2-3 (100 μg kg^−1^) and docetaxel (5 mg kg^−1^) resulted in substantially higher anti-metastatic activity than treatment with docetaxel only (Fig. 7b). Consistently, this combined treatment also further increased the survival rate of test mice in comparison with those only receiving each single treatment (Fig. 7c). Of note, treatment with low dosage of Q2-3 alone was already more effective than treatment with docetaxel, in terms of suppressing metastasis and prolonging survival; in combination with docetaxel, Q2-3 was even more effective (Fig. 7b,c). These data together suggest that *in vivo* administration of Q2-3 can confer strong complementary activity on the therapeutic activity of a clinically used anticancer drug, docetaxel, for suppression of metastatic tumour cell activities by regulating the tumour-associated microenvironment.

## Discussion

In previous studies, Q2-3 has been shown to cause cytotoxicity^13^ or G2/M cell cycle arrest^15^ in a number of tumour cell lines. Although significant *in vivo* antitumour activity of Q2-3 was not detected using a preliminary *in vivo* screening tool (hollow fibre assay)^13^, our present *in vivo* study clearly demonstrated that intravenous administration of Q2-3 (10–100 μg kg^−1^) for 3 weeks can considerably reduce or delay the growth of *in situ* mammary carcinoma tissue (Supplementary Figs 1 and 2). This apparent contradiction in findings on antitumour activity of Q2-3 may be due to a number of factors, including the difference in drug-delivery method of Q2-3 (intraperitoneal versus intravenous administration), experimental period or the space/nutrient limitation of the hollow fibre system versus the bonafide *in vivo* condition. By taking advantage of the 4T1-Luc2 and MDA-MD-231-Luc2 tumour resection models employed in this study, we could evaluate the pharmacological effect on tumour metastasis-related issues and change of tissue microenvironment in the target metastatic organ, which could be difficult to address using the hollow fibre system. Specifically, we demonstrated the potent suppressive activity of Q2-3 on metastasis of both mouse and human mammary carcinoma cells. In addition, we also revealed that Q2-3 can confer specific stimulatory activity on IL-25 expression form the surrounding TAFs (Fig. 3a,b). We hence believe that Q2-3 can effectively monitor and regulate the tumour microenvironment, resulting in efficacious anti-metastatic activities. We consider that this regulatory effect of Q2-3 has potential for translation into future clinical application for several reasons: Q2-3 stimulatory activity can be obtained at a very low dosage (20–100 μg kg^−1^ body weight), the phytochemical derivative can already be readily synthesized, and its molecular mode of action is specific, that is, it acts on the expression of an endogenous anticancer factor IL-25 against mammary tumour cells in ongoing metastasis.

Previously, Q2-3 was reported to exhibit an inhibitory activity for tubulin polymerization^13^. This finding may indicate that Q2-3 could share a similar molecular target in the cell; however, it could exhibit an opposite pharmacological activity to docetaxel, which is known as a stabilizator of microtubules^20^,^21^. Interestingly, our data also show that the use of Q2-3 in combination with docetaxel, a clinically used anticancer drug, can create a significant additive effect on the anti-metastatic activity (Fig. 6), suggesting that the control or regulation of the tumour microenvironment, an activity different from anti-mitotic division activity, may be employed as a separate or independent therapeutic approach for developing new class of anticancer therapeutics, including combinational treatment strategies. In the future, it will be worth investigating the comprehensive effect of the inhibitor and stabilizer activities of tubulin polymerization in regulating the malignancy, as well as non-malignant or normal cellular behaviours, in the context of the metastatic tumour microenvironment.

Although the control and regulatory mechanisms for IL-25 (IL-17E) expression in specific cell types, including type 2 helper T cells (Th2), macrophages, normal mammary epithelial cells and mast cells^9^,^22^, are not clear, some previous findings may in fact be related to the Q2-3-induced IL-25 expression by fibroblasts. First, a stimulatory activity of IL-25 was detected during lung inflammation and related cellular stress^23^,^24^. For our own study (Fig. 1), although Q2-3 exhibited a much lower cytotoxic effect on test non-malignant cells than on the mammary carcinoma cells, it apparently caused some stressful effect on test fibroblasts (WI38) and other non-malignant cells (M10). These findings may raise a hypothesis for the effect of Q2-3-induced cellular stress on the non-malignant cells in the tumour microenvironment, facilitating lung inflammation-mediated metastasis. In addition, IL-25 was also shown to induce some expression of various proinflammatory cytokines in lung fibroblasts^25^. Therefore, a positive-feedback mechanism may exist for IL-25 expressed and released specifically by pulmonary fibroblasts, which could further amplify the preliminary stimulation of Q2-3 in test lung tissues. Second, a number of transcription factors could be predicted to recognize the promoter region of human *IL25* gene, including p53, Glucocorticoid receptor-alpha and glucocorticoid receptor-beta, as revealed by *QIAGEN* (http://www.genecards.org/cgi-bin/carddisp.pl?gene=IL25#expression). Interestingly, with a study of our previous transcriptome analysis, we found that the expression level of *p53* gene was substantially decreased (∼40% decrease) in human lung fibroblasts (WI38), after co-cultivation with malignant tumour cells (MDA-MB-231) in 3D culture system. In contrast, Q2-3 treatment increased the expression of *p53* gene in both untreated (∼26% increase) and MDA-MB-231 cell co-cultured WI38 fibroblasts (∼92% increase). These results suggest that Q2-3 treatment may induce *p53* gene expression in tumour-associated fibroblasts, which has been shown to be suppressed by epithelial cancer cells^26^. On the other hand, although the transcription factor-binding sites for glucocorticoid receptor-alpha and glucocorticoid receptor-beta protein could also be predicted in both mouse and human IL-25 promoter region, their expression levels were not affected in WI38 cells after Q2-3 treatment. Taking these information together, it is plausible to hypothesize that Q2-3-induced IL-25 expression by fibroblasts could also be mediated by *p53*, a tumour suppressor gene with DNA repairing-related functions^27^,^28^. Third, in addition to the differential cytotoxicity of Q2-3 in treating malignant cells and non-malignant cells, our data also indicate that Q2-3 can effectively suppress the growth (≥60% inhibition) of human mammary tumour cells *in vitro* (≥0.5 μM) (Fig. 1b,c) and the metastasis of 4T1 cells into the lung tissue *in vivo* (≥20 μg kg^−1^) (Fig. 2a,b), at a relatively low concentration. These results together suggest that Q2-3 may result in a therapeutic effect via targeting to some specific cellar/protein target(s), which may be reflected by the different expression pattern observed between the malignant and non-malignant cells. Future identification of specific cellular target(s) for Q2-3 is critical for realizing the stimulatory mechanism of Q2-3-induced IL-25 expression by fibroblasts or normal epithelial cells present in the tumour-associated lung microenvironment.

Human IL-25 (IL-17E) was previously identified as a member of the IL-17 cytokine family and is secreted by type 2 helper T cells (Th2)^29^ and mast cells^22^, indicating that its function is closely related to specific immunity. Recently, IL-25 secreted by normal mammary epithelial cells was further shown to induce apoptosis of human breast cancer cells^9^, specifically, due to the high levels of IL-25R expressed by mammary tumour cells^9^. In the present study, we obtained data from *in vivo* and 3D co-culture systems, showing that IL-25 expression (Fig. 3) and secretion (Fig. 4a) from non-malignant fibroblasts also could be efficiently activated by Q2-3 treatment. On the basis of these results, we suggest that the *in vivo* mechanism for secretion of endogenous IL-25 can be conferred by different types of non-malignant cells. To reveal the pharmacological role of fibroblast-produced IL-25 in Q2-3-mediated anti-metastasis activities, the identification of specific intracellular target(s) of Q2-3 will be critical in future study and for clinical applications. On the other hand, for those 3T3 fibroblasts treated with Q2-3, the cells co-cultured with 4T1 metastatic tumour cells expressed a lower level of IL-25, compared with fibroblasts that were not co-cultured with any tumour cells (Fig. 3d). This result, however, was not consistent with the human fibroblasts (WI38 cells) tested in response to the tumour cell co-cultivation (Fig. 3e). We consider this will be an important issue in future study for evaluating the effect of metastatic tumour cells on expression and secretion activity of endogenous anticancer factors, specifically IL-25 (ref. 9), in the tumour microenvironment.

Human primary lung fibroblasts have been shown to constitutively express IL-25R (IL-17RB)^25^. In addition, IL-25/IL-25R signalling was shown to induce expression of proinflammatory cytokines, including CCL-5, CCL-11, CXCL-8, IL-6, GM-CSF and G-CSF in human lung fibroblasts^25^,^30^,^31^. In the present study, we demonstrate that IL-25 secretion activity of lung fibroblasts can be effectively induced by Q2-3. These findings raise the possibility that IL-25/IL-25R signalling in tumour-associated fibroblasts could induce a positive-feedback mechanism for IL-25 expression in the tumour microenvironment. On the other hand, some antigen-presenting cells, including macrophages and dendritic cells, have been indicated as the target cells for action of IL-25 (ref. 32). Specifically, macrophages have been indicated to serve as the cell type responsible for induction of IL-25/IL-33-mediated type 2 immunity^33^,^34^. Related to this finding, in our present study we also detected the expansion of pulmonary macrophage (CD206^+^ cells) in the lung tissue of Q2-3-treated mice (Supplementary Fig. 3), suggesting that IL-25 secretion in lung tissue may also facilitate the activation of specific types of immune cells and further participate in the anti-metastatic effect of Q2-3. In future study, the role of IL-25 in activating antigen-presenting cells may warrant evaluation, especially for revealing and developing the pharmacological basis of IL-25-mediated anti-metastatic activity and the development of cell-based cancer vaccines.

Recent studies on several ‘immune check point' molecules, such as programmed death 1 (PD-1)^35^,^36^,^37^,^38^ and cytotoxic T-lymphocyte-associated protein 4 (CTLA4)^39^,^40^,^41^ showed their antibodies or inhibitors can mediate suppression of T-cell immunity, which has been employed as a highly efficacious strategy for therapy of a spectrum of cancers^42^. These protein molecules are believed to negatively regulate the tumour immunogenicity. In this investigation, we showed that *in vivo* administration of Q2-3 at relatively low dosage (100 μg kg^−1^) can drastically improve the anti-metastatic activity of the clinically used chemotherapy drug, docetaxel (5 mg kg^−1^), when they are used in combination (Fig. 6). Because of the marked effect of the increased expression of IL-25 in Q2-3-mediated anti-metastasis, we would suggest that the IL-25/IL-25R interaction might also be considered as a new ‘immune check point' for activation of tumour immunogenicity. In other words, Q2-3-induced IL-25 expression in the tumour microenvironment may positively regulate and promote the immunogenicity of tumour cells to host immunity. Altogether, we consider that the specific pharmacological activity of Q2-3 on activating IL-25 secretion in the tumour microenvironment can upregulate a strong endogenous anticancer activity. Further studies are urgently needed to explore the clinical application of such Q2-3-mediated immune check point activation on tumour metastasis.

## Methods

### Compounds

Methyl(*E*)-3-[2-(3,4-dihydroxyphenyl)-7-hydroxy-3-methoxycarbonyl-2,3-dihydro-1-benzo furan-5yl]prop-2-enoate was prepared and characterized according to the method described previously^13^,^14^. Briefly, methyl caffeate was dimerized using silver oxide in the presence of anhydrous benzene and anhydrous acetone and the product was purified by silica gel column chromatography with ethyl acetate *n*-hexane as the eluent. After evaporation, a colourless amorphous solid was obtained (18.8%). ^1^H nuclear magnetic resonance (CDCl_3_, 400 MHz) *δ* p.p.m. 3.64 (3H, s), 3.66 (3H, s), 4.14 (1H, d, *J*=7.2 Hz), 5.87 (1H, d, *J*=7.2 Hz), 6.12 (1H, d, *J*=16.0 Hz), 6.61 (1H, dd, *J*=8.4,2.0 Hz), 6.69 (1H, d, *J*=8.4 Hz), 6.76 (1H, d, *J*=2.0 Hz), 6.90 (1H, br s), 6.93 (1H, br s), 7.43 (1H, d, *J*=16.0 Hz). For all experiments, the compound was freshly dissolved in DMSO as 50 mM stock solution and further dilutions were made in the complete medium.

### Cell lines

4T1, TS/A, 3T3, SKBR3, WI38, NHDF, MCF-7, MCF-10A and MDA-MB-231 cells were obtained from American Type Culture Collection (Manassas, VA, USA). All cell lines were tested negative for mycoplasma contamination. 4T1, 4T1-luc2 (that is, 4T1 cells transfected with a IF4γ promoter-driven luciferase gene), MDA-MB-231, MDA-MB-231-luc2 (that is, MDA-MB-231 cells transfected with a IF4γ promoter-driven luciferase gene), and SKBR3 and WI38 cell lines were kindly provided by Dr Pei-Wen Hsiao (Academia Sinica, Taipei, Taiwan)^43^,^44^. The MCF-10A cell line was provided by Dr Wen-Hwa Lee (Academia Sinica, Taipei, Taiwan). The NHDF cell line was provided by Dr Been-Huang Chiang (Institute of Food Science and Technology, National Taiwan University, Taipei, Taiwan). The 4T1 and MDA-MB-231-luc2 cells were maintained in RPMI-1640 (Invitrogen, Carlsbad, CA) complete medium supplemented with 10% fetal bovine serum (FBS), 100 μM non-essential amino acids and 100 μM sodium pyruvate. The stably transfected 4T1-luc2 cells were maintained in RPMI complete medium supplemented with 0.5% puromycin. 3T3, NHDF and TS/A cells were maintained in DMEM (Invitrogen, Carlsbad, CA) supplemented with 3.7 g l^−1^ sodium bicarbonate, 3.6 g l^−1^ HEPES and 10% FBS. MCF-7 cells were maintained in DMEM supplemented with 3.7 g l^−1^ sodium bicarbonate and 10% FBS. WI38 and M10 cells were maintained in MEM (Invitrogen, Carlsbad, CA) supplemented with 2.2 g l^−1^ sodium bicarbonate, 3.6 g l^−1^ HEPES, 1 × non-essential amino acid, 1 mM sodium pyruvate and 10% FBS. SKBR3 and MDA-MB-231 cells were maintained in DMEM/F-12 (Invitrogen, Carlsbad, CA) supplemented with 2 g l^−1^ glucose and 10% FBS. MCF-10A cells were maintained in DMEM/F-12 supplemented with 10 μg ml^−1^ insulin and 10% FBS. All culture media were supplemented with 100 μg ml^−1^ streptomycin, 100 units per ml penicillin and 2 mM L-glutamine. Cells were grown in a 5% CO_2_ incubator at 37 °C.

### Construction of the 3-dimensional co-culture system

To mimic the tumour microenvironment, a 3D co-culture system was employed for the maintenance of mammary tumour cells and tumour-associated fibroblasts in adjacent collagen gels. Briefly, rat tail collagen solution dissolved in acetic acid^45^,^46^ was neutralized by 1 N NaOH and then mixed with 10 × PBS at 4 °C in a ratio of 9:1. To construct solid tumour-like cell mass, mouse (4T1 or TS/A cells) or human (MCF-7 or MDA-MB-231 cells) mammary tumour cells in each correspondent culture medium (1 × 10^6^ cells per ml) were mixed with 4 mg ml^−1^ collagen solution in a ratio of 1:1. Four drops of collagen-tumour cell mixture (1 × 10^4^ cells per 10 μl per drop) were immediately and separately loaded on culture substratum in each well of a six-well plate. The plates were then turned over and kept in CO_2_ incubator at 37 °C for collagen gelation. To prepare foundation collagen gel (Fig. 3c), culture media were mixed with 4 mg ml^−1^ collagen solution in a ratio of 1:1 (final concentration: 2 mg ml^−1^). Aliquots of 1 ml foundation collagen solution were loaded on culture substratum and each was covered with collagen-tumour cell mixture. The culture plates were then kept in a CO_2_ incubator at 37 °C for another 10 min. To prepare the fibroblast-containing collagen gel, mouse (3T3 cells) or human (WI38) fibroblasts in each corresponding culture medium (3 × 10^6^ cells per ml) were mixed with 4 mg ml^−1^ collagen solution in a ratio of 3:1. One millilitre fibroblast-containing collagen solution (1 × 10^6^ cells per ml per well), and were immediately loaded on each solid foundation collagen gel. Finally, culture media for each fibroblast type were added onto the top layer and were suspended with test compounds for different drug treatments. After 24 or 72 h co-culture of tumour cells and fibroblasts, liquid-conditioned media were collected directly from each well for further tests.

For collection of total protein from fibroblasts in 3D collagen gel, a layer of fibroblast-containing collagen gel was separated from foundation gel, by using a pipet tip to smoothly scrape the collagen layer. Each collected fibroblast-containing collagen gel was then minced in an eppendorf tube using scissors and dissolved in 1 ml Trizol (Invitrogen) or 1 ml Tissue Protein Extraction Reagent (Thermo), for extraction of total RNA and cellular protein, respectively.

### MTT assay

M10, WI38, SKBR3 and MDA-MB-231 cells (1 × 10^5^ cells per ml) dispensed in 96-well plates were incubated with vehicle or test compounds (Q2-3) for 24 h or 72 h in corresponding basal medium in a 5% CO_2_ incubator. To evaluate the effect of secreted IL-25 from Q2-3-treated fibroblasts on growth activity of mammary tumour cells, 4T1 and MDA-MB-231 cells (1 × 10^4^ cells per ml) dispensed in 96-well plates were incubated with control medium or different fibroblast-conditioned media (200 μl ml^−1^) which was changed daily for 5 days. All treatments were performed in triplicate cell cultures. The growth activity of cells was assayed using a 3-(4, 5-dimethythiazol-2-yl)-2, 5-diphenyl tetrazolium bromide (MTT; Sigma-Aldrich) colorimetric method. The absorbance at 570 nm (A570) was measured using a multiwall scanning spectrophotometer.

### Mice

For this study, female BALB/c mice and nude mice (BALB/cAnN.Cg-*Foxnl*^*nu*^/CrlNarl) age 6–8 weeks were purchased from the National Laboratory Animal Breeding and Research Center, Taipei, Taiwan. Test mice were maintained in a laminar airflow cabinet kept at 24±2 °C and 40–70% humidity with 12-h light/12-h dark cycles under specific pathogen-free conditions. All manipulation and experimental protocols involving animals were approved by the Institutional Animal Care and Utilization Committee (IACUC) of Academia Sinica, Taipei.

### 4T1-Luc2 and MDA-MD-231-Luc2 tumour resection model

BALB/c mice were subcutaneously injected with 4T1-Luc2 cells (5 × 10^5^ cells per 100 μl PBS per mouse) into the fourth mammary fat pad under isoflurane anaesthesia^47^. Tumour growth was monitored by measuring the tumour volume according to the formula: volume=length × (width)^2^/2. After tumours were established (180–200 mm^3^) on day 14, test mice were divided into different groups (8 mice per group) and subjected to different treatments. At 15 days post tumour cell implantation, primary 4T1 tumours *in situ* were surgically removed by a tumour resection process. For drug treatment, mice were administered with different agents, including vehicle–control (PBS), mouse IL-25 protein (10 μg kg^−1^, ProSpec, Vineland, NJ), Q2-3 (2, 20 or 100 μg kg^−1^) or docetaxel (2 mg kg^−1^; Sigma-Aldrich), by intravenous injection for 3 weeks (3 injections per week) post tumour resection. For *in vivo* neutralization of mouse IL-25 activity, mice were treated intraperitoneally with either 100 μg anti-mouse IL-25 (IL-17E) antibody (clone 35B; Biolegend) or isotype control rat IgG (100 μg; rat, IgG1κ; Biolegend) at days 0, 3, 6, 9, 12, 15, 18 post tumour resection. To detect metastasis of human MDA-MD-231-Luc2 tumour cells, nude mice were injected subcutaneously with 5 × 10^5^ MDA-MD-231-Luc2 cells into the mammary fat pad under isoflurane anaesthesia. After tumours were established (250–350 mm^3^) on day 24, test mice were divided into different groups (8 mice per group) and subjected to different treatments. At 25 days post tumour cell implantation, primary MDA-MD-231-Luc2 tumours *in situ* were surgically removed by a tumour resection process. For drug treatment, mice were administered with PBS (vehicle–control), Q2-3 (100 μg kg^−1^), docetaxel (5 mg kg^−1^) or Q2-3 (100 μg kg^−1^)+docetaxel (5 mg kg^−1^), by intravenous injection for 3 weeks (3 injections per week) post tumour resection. To monitor the progression of mouse (4T1) or human (MDA-MD-231) metastatic tumours, test mice treated with different drugs/agents were compared for their tumour metastatic activity and survival rate aftern another 8 weeks. Bioluminescence signals from the 4T1-luc2 tumour cells in test mice were analysed using a non-invasive IVIS imaging system (Calipers, Hopkinton, MA) after intraperitoneal injection of 150 mg kg^−1^ D-luciferin (NanoLight technology, Pinetop, AZ).

### Immunofluorescence staining

Lung tissue specimens obtained from each tumour-resected mouse were fixed with 4% formalin and embedded in paraffin. For histological comparison, 6-μm-thick tissue sections were made and stained with hematoxylin and eosin (H&E). For immunofluorescence staining, fixed tissue sections were initially immersed in boiling sodium citrate buffer (0.01 M sodium citrate buffer, pH 6.0) for 30 min. Lung tissue sections were blocked with 5% nonfat milk, and incubated with anti-FSP-1 antibody (1:200 dilution; Millipore; catalogue number: 07-2274), FITC-conjugated anti-CD206 antibody (1:200 dilution; Biolegend; catalogue number: 141703) or PE-conjugated anti-IL-25 antibody (1:100; R&D; catalogue number: IC12991P) in 1% nonfat milk for 1 h at room temperature. Sections were then washed with PBS containing 0.1% Tween 20. To detect primary antibodies, some sections were incubated with FITC–conjugated anti-rabbit-IgG (1:200; Jackson Immunoresearch, West Grove, PA; catalogue number: 111-097-103) for FSP-1. 4′,6-Diamidino-2-phenylindole dihydrochloride (1 μg ml^−1^; Sigma-Aldrich) was used to stain the nuclei. Fluorescence microscopy evaluation of immunostained tissue sections was performed using a Zeiss Axiovert 200 M microscope (Carl Zeiss, Heidelberg, Germany). Images were captured with a digital camera (Orca ER, Hamamatsu) and processed using Axiovision 4.6.3 (Carl Zeiss).

### Antibody-mediated depletion of IL-25 in conditioned medium

To pull down and deplete IL-25 protein molecules in 3T3 or MDA-MB-231 cell conditioned media, the Dynabeads antibody coupling kit (Life Technology; 14311D) was used according to the manufacturer's recommendations, yielding 10 mg ml^−1^ anti-IL25 antibody (ProteinTech, Chicago, IL; catalogue number: 11353-1-AP)-coupled beads. The rabbit IgG (ProteinTech; catalogue number: 30000-0-AP) was used as an isotype control antibody. After antibody-coupling reaction, each conditioned medium (4 ml) was reacted with 2 mg antibody-coupled beads on a roller at room temperature for 1 h. The CM was then placed on a magnet for 1 min allowing the beads to be collected on the tube wall. The antibody-pulled down proteins from 3T3-CM, NHDF-CM or WI38-CM, were detected for the content of secreted IL-25 protein in each conditioned medium using western blot analysis. For some tests, the supernatants were collected and used for treating mouse or human mammary tumour cells.

### IL-25R siRNA treatment

MDA-MB231 and MCF-7 cells were seeded in six-well plate at 10^5^ cells per well for 24 h before transfection. siRNAs used for knockdown of human IL-25 receptor (IL25-RB) were purchased from Biotools (Taiwan) as follows: IL-17RB-homo-448 (IL-25R-1 siRNA; sequence: 5′-AUUAGGAAUAUUAUGGGCCTT-3′); IL-17RB-homo-519 (IL-25R-2 siRNA; sequence: 5′-AUUAUGUGGUCUAGGCAGCTT-3′); IL-17RB-homo-956 (IL-25R-3 siRNA; sequence: 5′-ACAUUAGAUAGAUCCCUGCTT-3′); negative control (Neg; sequence: 5′-ACGUGACACGUUCGGAGAATT-3′). At the beginning of transfection, each test IL25R siRNA oligomers (100 pmol) was diluted in 250 μl Opti-MEM I Reduced Serum Medium. Aliquots of 5 μl Lipofectamine 2000 transfection reagent (Invitrogen) were diluted with 250 μl Opti-MEM I Reduced Serum Medium. Diluted oligomers were mixed gently with the diluted Lipofectamine 2000 and incubated for 20 min at room temperature. The oligomer–Lipofectamine 2000 complexes were subsequently added to each well containing cells and medium. Cells were incubated at 37 °C for 72 h until test cells were ready to be treated with IL25 cytokine or a different CM.

### Western blot assay

Cell lysates of 3T3, WI38, NHDF, MDA-MB-231, MCF-10A and MCF-7 or the IL-25-pulled down protein lysates from 3T3, WI38 or NHDF CM were resolved by SDS–PAGE using 8, 10 or 15% stepwise gels. The resolved proteins were transferred onto a PVDF membrane (Novex, San Diego, CA) and blotted with anti-IL-25 (1:1,000 dilution; ProteinTech; catalogue number: 11353-1-AP), anti-IL-25R (1:2,000 dilution; ProteinTech; catalogue number: 20673-1-AP), anti-Caspase-3 (1:4,000 dilution; Abcam; catalogue number: ab13847), anti-Caspase-8 (1:4,000 dilution; Cell Signaling; catalogue number: #9746), or anti-β-actin antibody (1:10,000 dilution; Abcam; catalogue number: #8227). The membrane was blocked with 5% nonfat dry milk in PBST buffer (PBS containing 0.1% Tween 20) for 60 min at room temperature. Blotted membranes were then incubated overnight at 4 °C with specific, commercially available antibodies. Loading of equal amounts of protein was assessed using the mouse β-actin protein as a reference. The blots were rinsed three times with PBST buffer for 5 min each. Washed blots were incubated with HRP-conjugated secondary antibody (1:10,000 dilution; Abcam) and washed again three times with PBST buffer. The transferred proteins were visualized with an enhanced chemiluminescence detection kit (Amersham Pharmacia Biotech, Buckinghamshire, UK). Quantification of protein in luminescent bands was performed using ImageJ software.

### Flow cytometry assays

For detection of MDSCs, cells from mouse lung tissue in each group were collected and stained for 30 min at 4 °C with antibodies against specific cell markers, including FITC-conjugated anti-mouse CD11b (Biolegend; catalogue number: 301403), APC-Cy7 conjugated anti-mouse Ly-6C (Biolegend; catalogue number: 128026) and PE-conjugated anti-mouse Ly-6G (Biolegend; catalogue number: 127618). The percentages of monocytic and granulocytic MDSCs were gated on CD11b^+^Ly-6C^+^ cells and CD11b^+^ Ly-6G^+^ cells, respectively^48^. For quantification of IL-25-expressing fibroblasts, individual cells isolated from mouse lung tissue were stained with Alexa Fluor 647-conjugated anti-ER-TR7 (Novus, USA; catalogue number: NB100-64932AF647) and anti-FSP-1 (Millipore; catalogue number: 07-2274) primary antibody followed by FITC-conjugated secondary antibody (Abcam; catalogue number: ab97050). PE-conjugated anti-mouse IL-17E antibody (R&D; catalogue number: IC13991P) was used for staining of intracellular IL-25 molecules in lung tissue. The percentage of IL-25-expressing fibroblasts was gated for FSP-1^+^ ER-TR7^+^ cells. Flow cytometry was performed on a FACS LSR II (BD, Netherlands) machine at the Agricultural Biotechnology Research Center (ABRC) in Academia Sinica.

### Statistical analysis

Statistical analysis was performed using an unpaired, two-tailed Student's *t*-test. Statistical analyses were conducted with GraphPad Prism 5.0 (GraphPad Software). Differences in tumour metastasis or mouse survival rate were determined by a log-rank (Mantel-Cox) test of the Kaplan–Meier curves. All statistical tests were two-sided. A P value of <0.05 was considered significant (*P<0.05; **P<0.01; ***P<0.001; NS, not significant).

## Additional information

**How to cite this article**: Yin, S. Y. *et al.* Induction of IL-25 secretion from tumour-associated fibroblasts suppresses mammary tumour metastasis. *Nat. Commun.* 7:11311 doi: 10.1038/ncomms11311 (2016).

## Supplementary Material

Supplementary Information Supplementary Figures 1-4
