# Erratum: Induction of IL-25 secretion from tumour-associated fibroblasts suppresses mammary tumour metastasis

**DOI:** 10.1038/ncomms11909

**Published:** 2016-06-03

**Authors:** Shu-Yi Yin, Feng-Yin Jian, Yung-Hsiang Chen, Shih-Chang Chien, Mao-Chih Hsieh, Pei-Wen Hsiao, Wen-Hwa Lee, Yueh-Hsiung Kuo, Ning-Sun Yang

Nature Communications 7: Article number: 11311 10.1038/ncomms11311 (2016); Published: 04182016; Updated: 06032016

The author Yueh-Hsiung Kuo was incorrectly omitted from the list of corresponding authors in this Article. The correct information for correspondence is: ‘Correspondence and requests for materials should be addressed to Y.-H.K. kuoyh@mail.cmu.edu.tw) or to N.S.Y. nsyang@gate.sinica.edu.tw)'.

