# Supplementary material for: Induction of IL-25 secretion from tumour-associated fibroblasts suppresses mammary tumour metastasis
Source: Nat Commun. 2016 Apr 18;7:11311. doi: 10.1038/ncomms11311 (PMC4837478; doi:10.1038/ncomms11311)
Supplement: Supplementary Information — Supplementary Figures 1-4 [file ncomms11311-s1.pdf]

Supplementary Figure 1:

a

0 week after drug administration

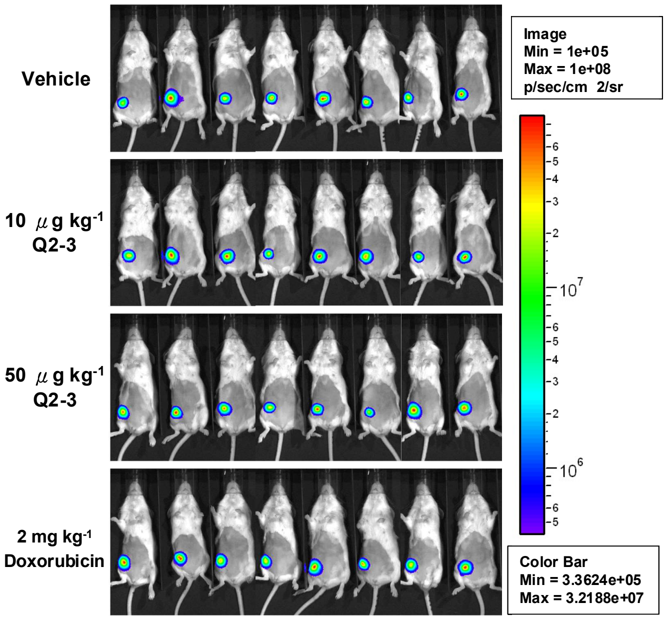

3 weeks after drug administration

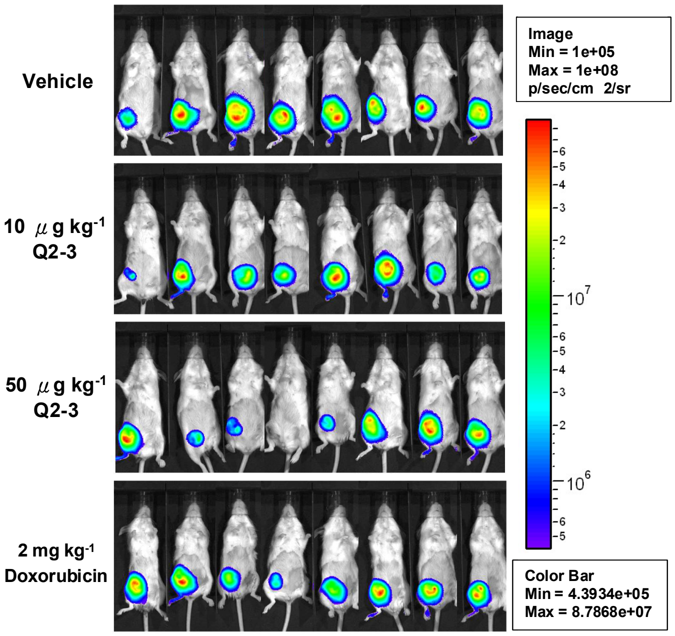

b

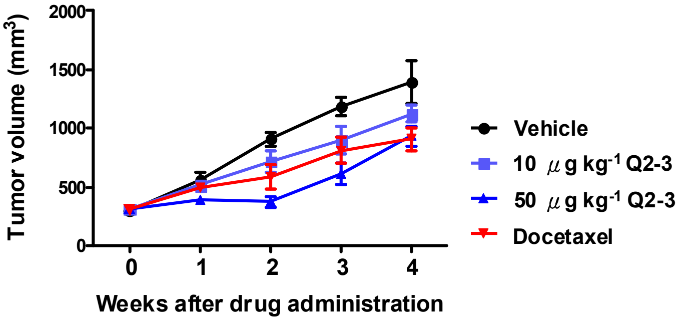

"Vehicle" v.s "10 μg kg^{-1} Q2-3" $P > 0.05$, n.s

"Vehicle" v.s "50 μg kg^{-1} Q2-3" $P < 0.05$, *

c

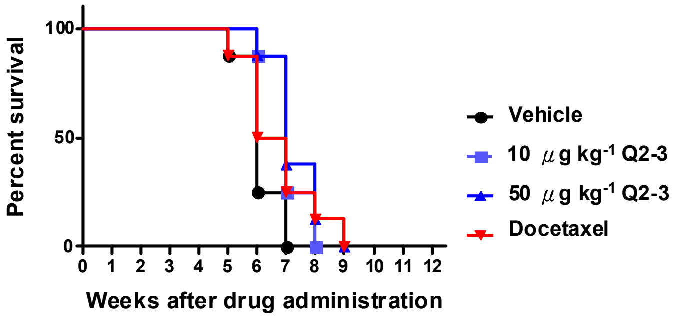

"Vehicle" v.s "10 μg kg^{-1} Q2-3" $P > 0.05$, n.s

"Vehicle" v.s "50 μg kg^{-1} Q2-3" $P < 0.05$, *

Supplementary Figure 1. In vivo treatment of Q2-3 suppresses the growth of mouse (4T1) mammary tumor *in situ*. (a) Representative bioluminescent images of mice (n = 8 per group) with 4T1 mammary carcinoma *in situ,* after in vivo treatment with PBS (Vehicle, 0.1% DMSO in saline), Q2-3 (10 or 50 μg kg^{-1}; 3 injections week^{-1}), docetaxel (2 mg kg^{-1}) for 0 and 3 weeks. (b) Quantification of tumor volume (mm^2) in mice, as determined from the length (A) and width (B) of test tumors by the formula $V = A \times B^2/2$. (c) Survival of test mice after different treatments. n.s, no significant. *, significance of $P < 0.05$ (Kaplan–Meier results were analyzed by log-rank test), when compared with the mice in vehicle control group. Similar results were obtained from two independent experiments.

Supplementary Fig 2:

a

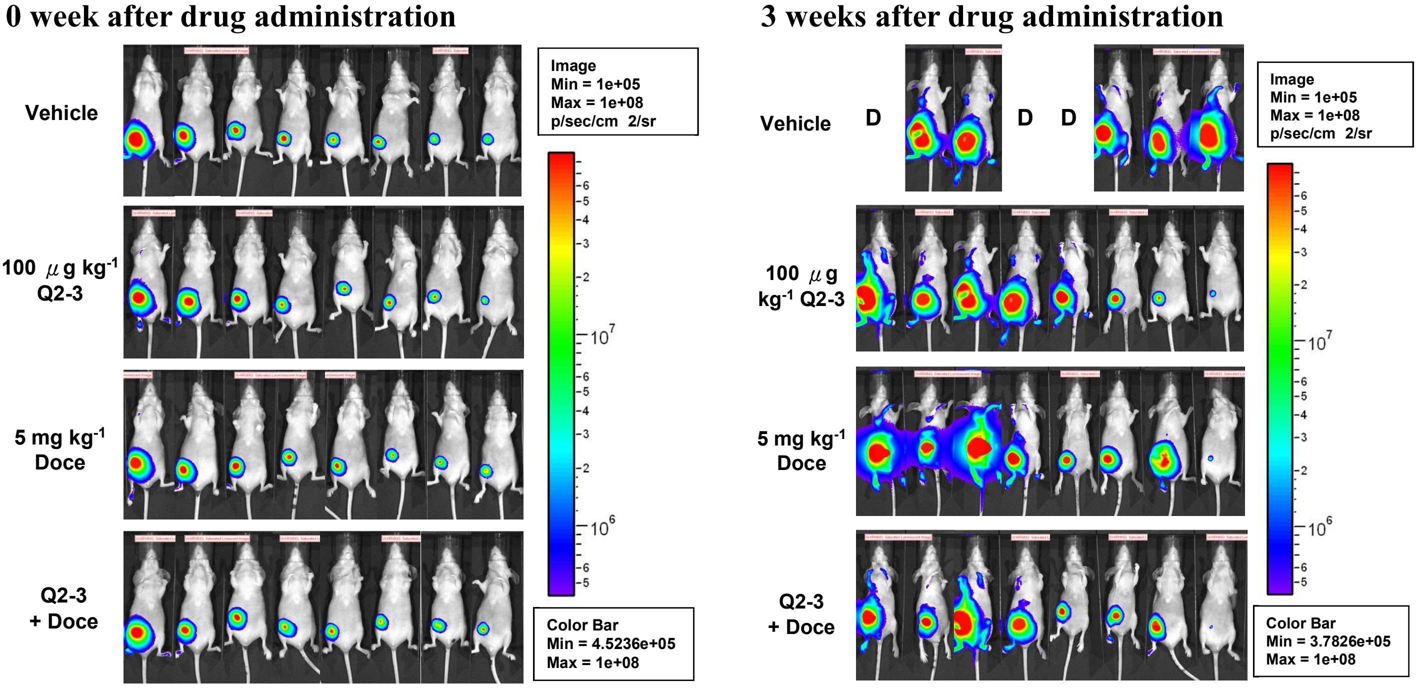

b

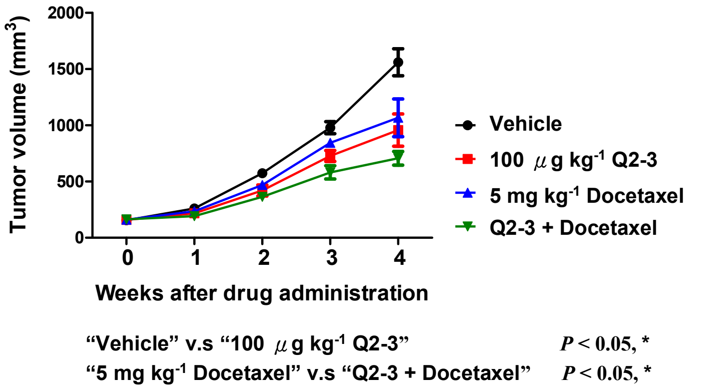

c

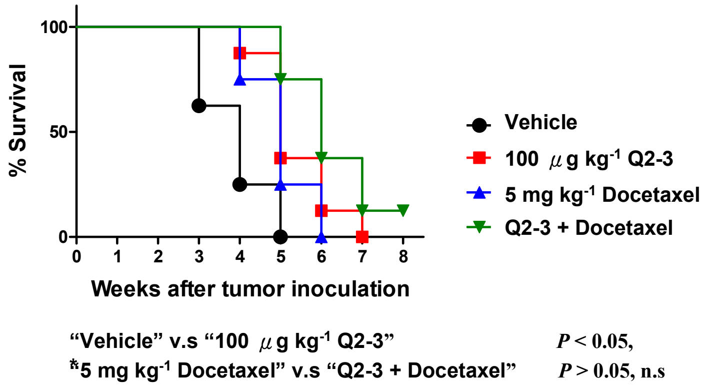

Supplementary Figure 2. In vivo treatment of Q2-3 suppresses the growth of human (MDA-MB-231) mammary tumor *in situ*. (a) Representative bioluminescent images of mice (n = 8 per group) with MDA-MB-231 mammary carcinoma *in situ,* after in vivo treatment with PBS (Vehicle, 0.1% DMSO in saline), Q2-3 (100 μg kg^{-1}; 3 injections $week^{-1}$), docetaxel (5 mg kg^{-1}) or co-treatment with docetaxel and Q2-3 for 0 and 3 weeks. Labelling with "D" in photograph denotes the mice were died before image taken. (b) Quantification of tumor volume (mm^2) in mice, as determined from the length (A) and width (B) of test tumors by the formula $V = A \times B^2/2$. (c) Survival of test mice after different treatments. n.s, no significant. *, significance of $P < 0.05$, as indicated comparisons. Similar results were obtained from two independent experiments.

Supplementary Fig 3:

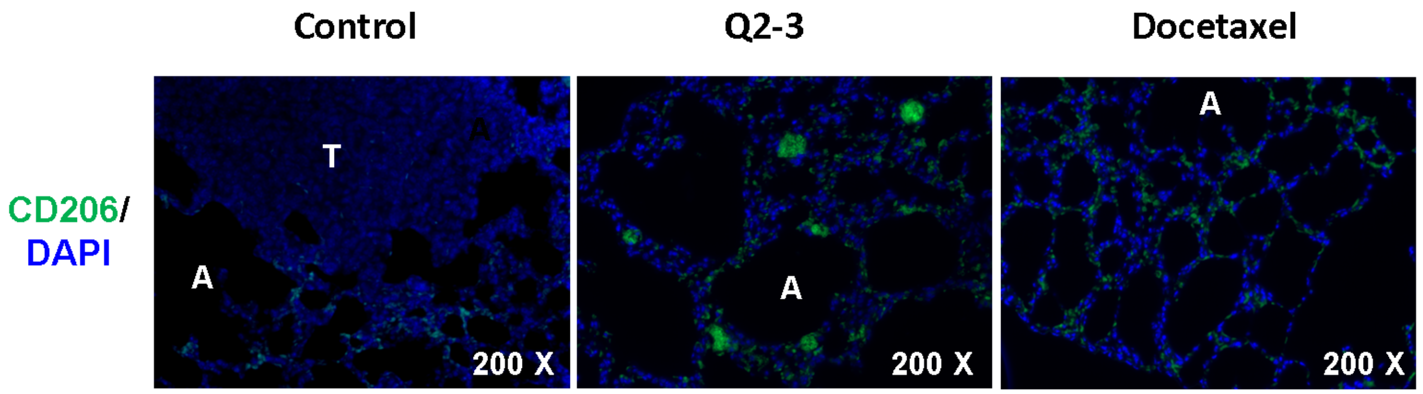

Supplementary Figure 3. Q2-3 treatment significantly induces the expansion of pulmonary macrophages in lung tissues of Q2-3-treated mice. The presence of $CD206^{+}$ cells (green) indicates the clustering of microphages in lung tissue of Q2-3-treated mice, rather than in control or docetaxel-treated mice.

Supplementary Fig 4

Figure 3d

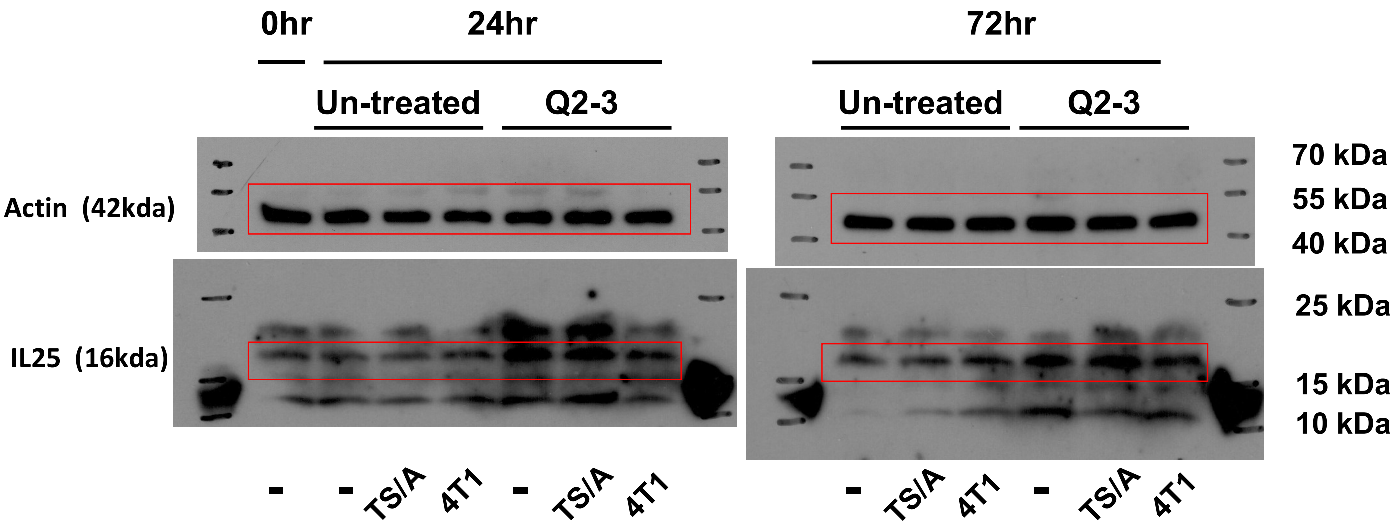

Figure 3e

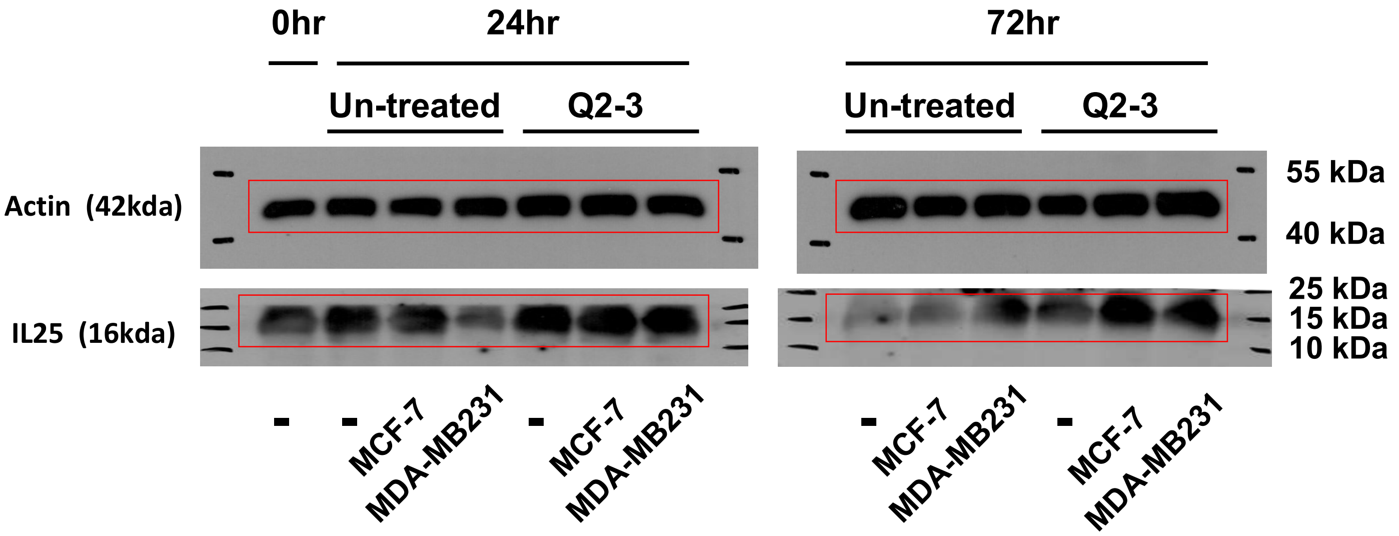

Supplementary Figure 4. Uncropped scans of four key western blot analyses on IL-25 expression from Q2-3-treated fibroblasts. Areas shown in Figure 3d and 3e are enclosed in red boxes and the molecular weight markers are shown on the right panel.
